# Supplementary material for: Microglial cyclooxygenase-1 modulates cerebral capillary basal tone in vivo in mice
Source: Nat Commun. 2025 Jul 1;16:5704. doi: 10.1038/s41467-025-60753-x (PMC12216979; doi:10.1038/s41467-025-60753-x)
Supplement: Supplementary file 1 — Supplementary Information [file 41467_2025_60753_MOESM1_ESM.pdf]

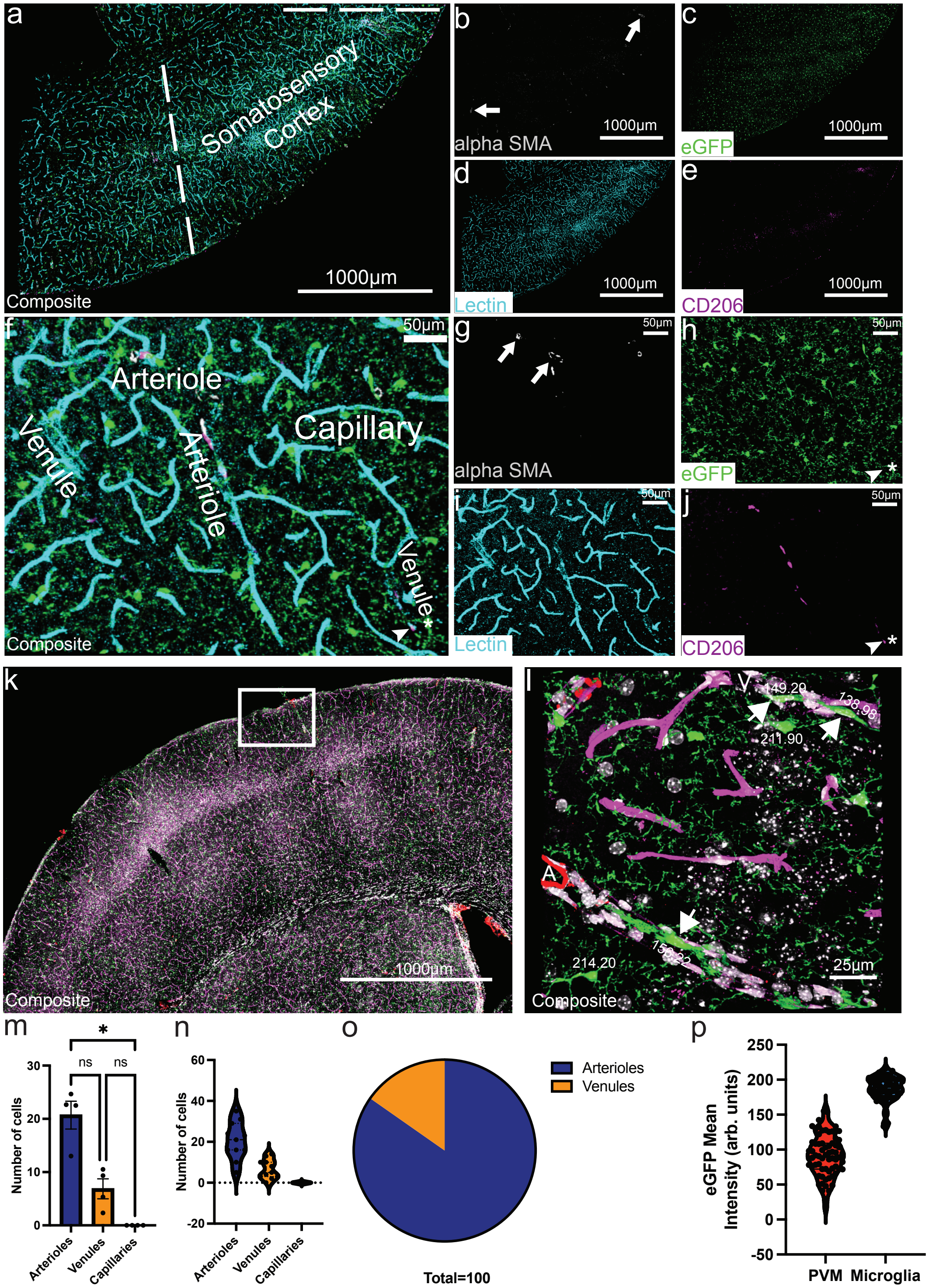

Supplementary Figure 1- Distribution of microglia and CD206+ meningeal and perivascular macrophages along the vascular tree a) A composite 10X tiled image showing the

somatosensory cortex in a CX3CR1-eGFP mouse. Dashed lines indicate the region of the somatosensory cortex where analyses were conducted. **b-e)** Individual channels of the composite image shown in **a**. The arrows in **b** indicate the location of alpha smooth muscle actin labeling (white) on arterioles. **f)** A 20X image of somatosensory cortex showing both alpha-smooth muscle actin positive arterioles and alpha-smooth muscle actin negative venules. The arrow indicates the location of alpha smooth muscle actin labeling on arterioles. **g-j)** 20X images of the same image in **f**, but showing the individual channels. The asterisk and arrowhead in **h and j** indicates an $eGFP^{+}$:$CD206^{-}$ microglia and a $CD206^{+}$ perivascular macrophage (magenta), respectively. **k)** Tiled volumetric 3D reconstruction from 20X confocal z-stacks showing CX3CR1-eGFP myeloid cells along lectin-labeled (cyan) α-smooth muscle actin (SMA) arterioles or αSMA negative venules. The white box indicates the zoomed in region shown in **l**. **l)** Volumetric 3D reconstruction from a 63X confocal z-stack showing an arteriole in the bottom left, indicated by A, and a venule in the top right, indicated by V. The arrows indicate nucleated perivascular macrophages, and the number above $eGFP^{+}$ cells are their mean intensity values, showing that we are counting nucleated CX3CR1-$eGFP^{low}$ perivascular macrophages. **m)** Bar graph quantifying the number of perivascular macrophages at arterioles (blue bar), venules (orange bar), and capillaries (white bar). n=11 tiled images (as in k) across 4 mice. Friedman test with Dunn's multiple comparison test. Adjusted p-values: Arterioles vs. Venules, $p<0.4719$; Arterioles vs. Capillaries, $p<0.0140$; Venules vs. Capillaries, $p<0.4719$. **n)** Violin plot showing the raw data used to generate the bar graph in **m**. **o)** pie chart showing the frequency of nucleated perivascular macrophages at arterioles (blue) and venules (orange). **p)** Violin plot used to generate the bar graph of perivascular macrophages and microglia mean eGFP intensity shown in Figure 1l. Data presented as mean ± s.e.m. Source data are provided as a source data file.

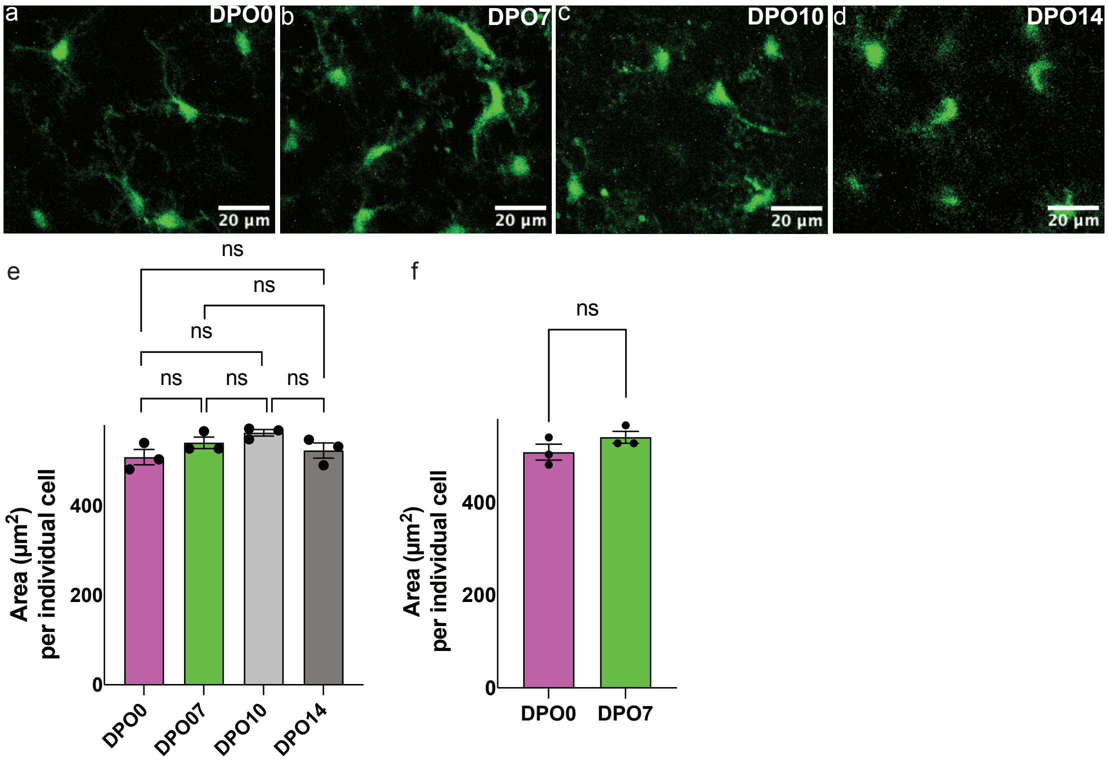

Supplementary Figure 2- Characterization of microglial reactivity following cranial window surgery in male mice Before commencing with 2Phatal experiments, we first wanted to characterize the timeline of microglial reactivity following cranial window implantation. Thus, we implanted cranial windows in male CX3CR1-eGFP mice and performed two-photon imaging at baseline, day post-operation (DPO) 7, DPO10, and DPO14. **a-d)** Maximum intensity projections of microglia at **a)** baseline (DPO0) (far left), **b)** DPO7, **c)** DPO10, and **d)** DPO14. **e)** bar graph comparing area of individual microglia in male mice at DPO0, DPO7, DPO10, and DPO14. n=9 microglia across 3 mice. Kruskal-Wallis test with Dunn's multiple comparisons test. Adjusted p-values: DPO0 vs. DPO7, p>0.9999; DPO0 vs. DPO10, p<0.1033; DPO0 vs. DPO14, p>0.9999; DPO7 vs. DPO10, p<0.8420; DPO7 vs. DPO14, p>0.9999; DPO10 vs. DPO14, p<0.4173. **f)** bar graph comparing area of individual microglia in male mice at DPO0 and DPO7. n=9 microglia across 3 mice. Two-tailed Mann Whitney test, p=0.4000. For all graphs, individual data points represent the averages of three randomly selected microglia area measurements from one mouse, so three dots equates to three mice. Data presented as mean ± s.e.m. LUTs have been adjusted in shown images. Source data are provided as a source data file.

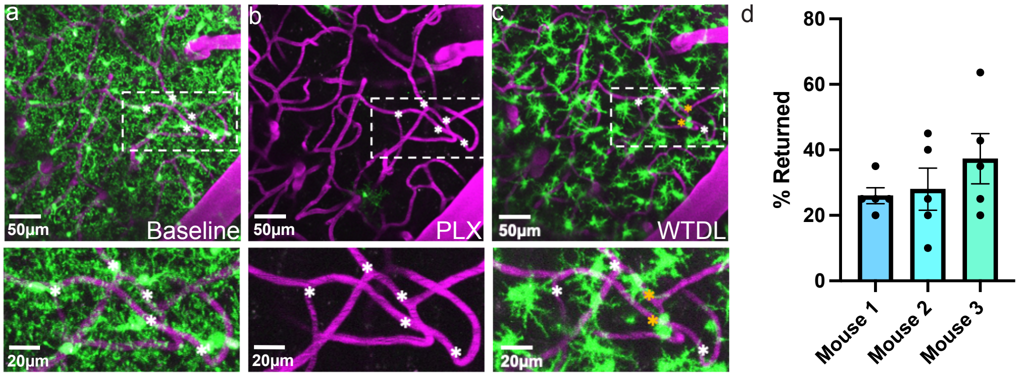

Supplementary Figure 3- Only a subset of vascular locations are repopulated by microglial following PLX3397 withdrawal. a-c) Maximum intensity projections of somatosensory cortex in a cranial-windowed CX3CR1-eGFP mouse at baseline **(a),** day 8 of PLX3397 administration **(b)**, and day 8 of PLX3397 withdrawal **(c)**. The white box in each image indicates the location of the zoomed in location shown at the **bottom**, which was used for the data analysis shown in **d**. **d)** Bar graph quantifying the percentage of vascular locations occupied at baseline by microglia that are also occupied at day 8 PLX3397 withdrawal. Data presented as mean ± s.e.m. LUTs have been adjusted in shown images. Source data are provided as a source data file.

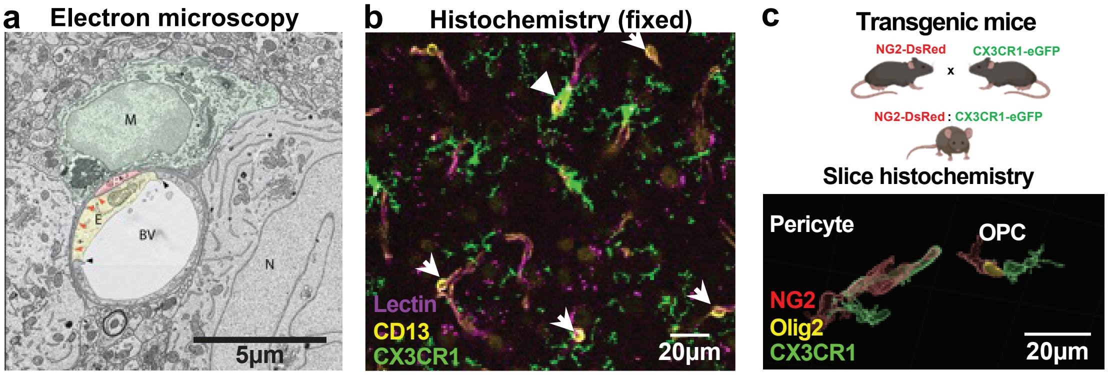

Supplementary Figure 4-Microglia associate with pericytes a) Electron micrograph showing microglia (pseudocolored green) juxtaposed to a pericyte (pseudocolored pink) and an endothelial cell (pseudocolored yellow). **b)** Confocal image of a cortical brain slice from a CX3CR1-eGFP mouse stained with CD13 to label pericytes and lectin to label blood vessels. Arrows identify pericytes on capillaries and arrowhead identify a pericyte with an associated microglia along the capillary. We refer to these as a “mericyte”. **c)** Mating scheme to generate double transgenic NG2-DsRed and CX3CR1-eGFP mice. NG2 labels both pericytes and OPCs that can be distinguished immunohistochemically by expression of Olig2 (expressed by OPCs but not pericytes).

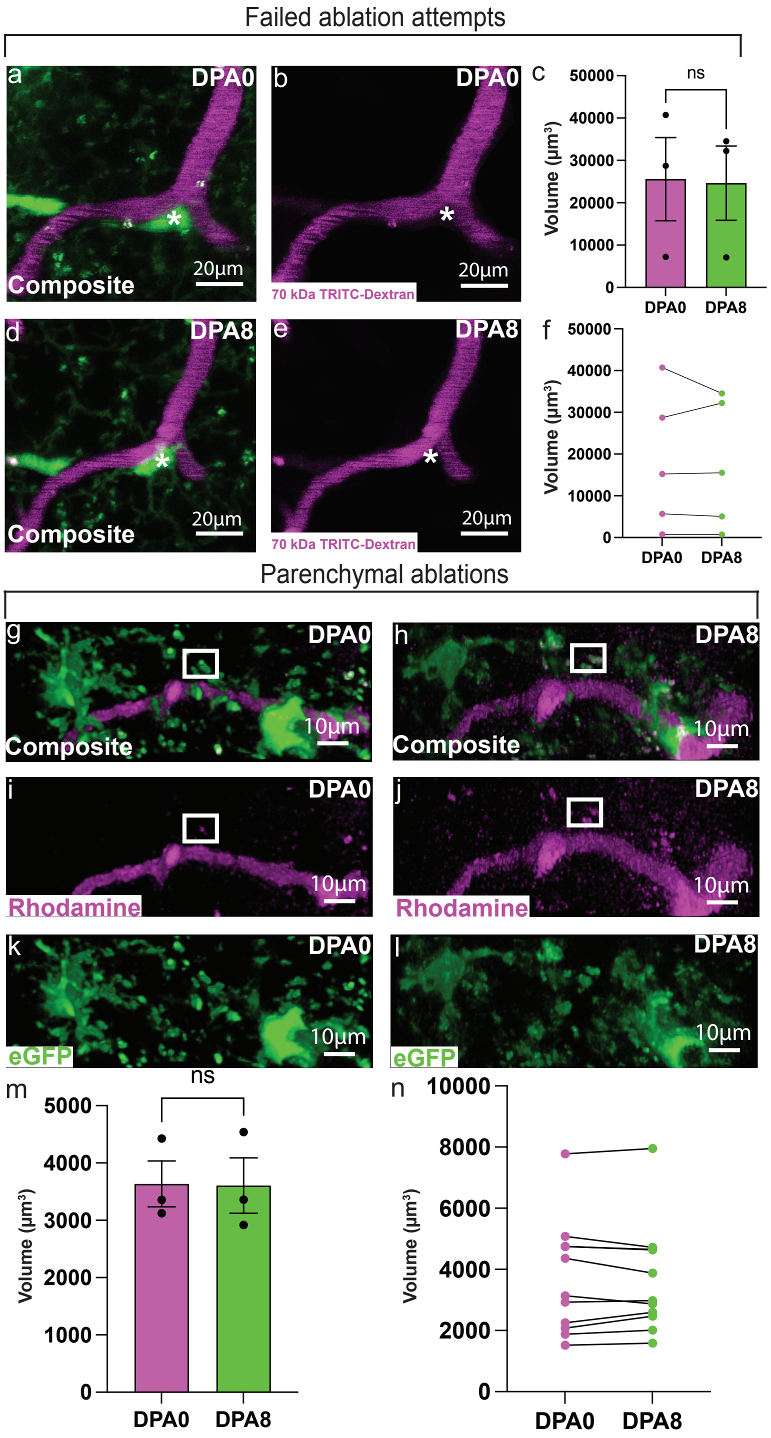

Supplementary Figure 5-Failed 2Phatal attempts and parenchymal laser-stimulation alone result in no change to capillary volume a-b) 3D volumetric reconstruction showing a CAM-

laden capillary region at day post-ablation 0 (DPA0) in somatosensory cortex of CX3CR1-eGFP mice, where **a** is the composite image and **b** just the TRITC-Dextran channel (magenta). The asterisk indicates an attempted 2Phatal ablation. **c)** Bar graph comparing the change in volume from baseline (DPA0) to DPA8 following a failed ablation attempt. Two-tailed ratio paired t-test, $p<0.5683$. n=5 capillaries/3 mice. **d and e)** 3D volumetric reconstructions of the same region shown in **a and b**, but at 8 days following a failed ablation attempt (DPA8). **d** shows the composite image and **e** just the TRITC-Dextran channel. **f)** a before and after plot showing the values used to generate the bar graph in **c**. **g-h)** 3D volumetric reconstructions showing a capillary region in CX3CR1-eGFP mice where the white square indicates a region of parenchymal space ablated with laser-power identical to that used in 2Phatal CAM experiments. **g** shows the composite image at baseline, and **h** the composite image of the same location at DPA8. **i)** 3D volumetric reconstruction showing the same capillary region at baseline as shown in **g,** but just the Rhodamine channel (magenta). **j)** 3D volumetric reconstruction showing the same region as that in **h**, but just the Rhodamine channel. **k)** 3D volumetric reconstruction showing the same region as that in **g**, but just the eGFP channel. **l)** 3D volumetric reconstruction showing the same region as that in **h**, but just the eGFP channel. **m)** a bar graph comparing change in capillary volume from baseline to DPA8 following parenchymal ablations. Two-tailed ratio paired t-test, $p<0.6653$. n=11 capillaries/3 mice. **n)** Before-and-after-line plot showing the raw data used to generate the bar graph in **m.** Data presented as mean ± s.e.m. LUTs have been adjusted in shown images. Source data are provided as a source data file.

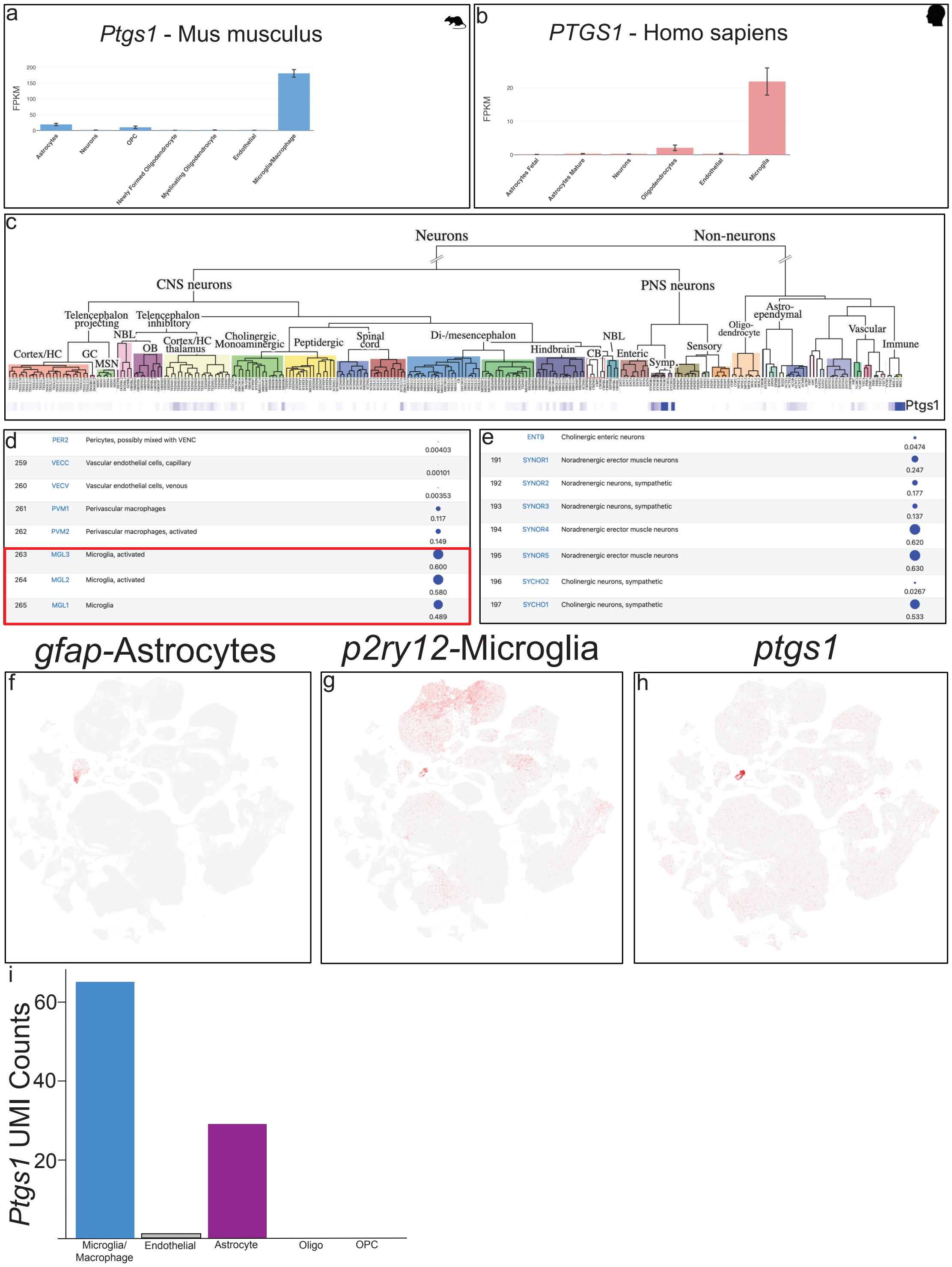

Supplementary Figure 6- Ptgs1 (COX1) transcriptional expression is highest in microglia compared to other cells of the central nervous system a-b) Bar graphs from the Barres lab

RNAseq database[43-44] showing Ptgs1 FPKM values by CNS cell type in mice, as in a, and humans- as in b. **c**) dendrogram from the Linnarsson lab's atlas of the adolescent mouse brain[45] describing identified cell types from their dataset. **d-e**) circle plots from the same atlas in c showing transcriptional expression values of Ptgs1 by the highest expressors in this dataset, including microglia and noradrenergic erector muscle neurons. **f-h**) Uniform Manifold Approximation and Projections (UMAPs) from the Allen Institute[46] showing the overlap or lack thereof between gfap astrocytes (left), p2ry12 microglia, and Ptgs1. **i**) bar graph from that same dataset showing Ptgs1 unique molecular identifier (UMI) counts in microglia (blue bar), endothelial cells (gray bar), and astrocytes (magenta bar). Source data are provided as a source data file.

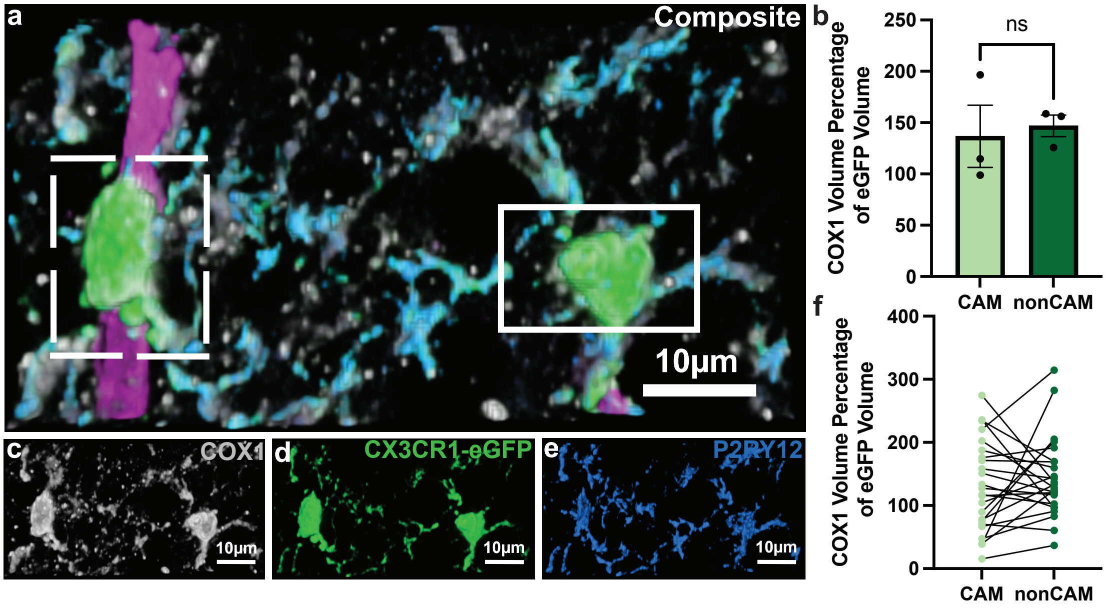

Supplementary Figure 7- Capillary-associated microglia (CAM) cyclooxygenase-1 (COX1) levels are no different from non-CAM COX1 levels. a, c-e) Three-dimensional reconstructions from CX3CR1-eGFP mice showing cyclooxygenase-1 (COX1) levels in P2RY12+ CAM (dashed line box) versus nonCAM (continuous line box), as in a. Just the COX1 channel (grey) is shown in c, the eGFP channel (green) in d, and P2RY12 channel (blue) in e. **b)** bar graph quantifying the percentage of COX1 volume that occupies total CX3CR1-eGFP microglia cell body volume. n=24 CAM and neighboring non-CAM measurements over 3 mice. Two-tailed paired t-test, $p<0.7581$. **f)** the raw data used to generate the bar graph shown in b. LUTs have been adjusted. Source data are provided as a source data file.

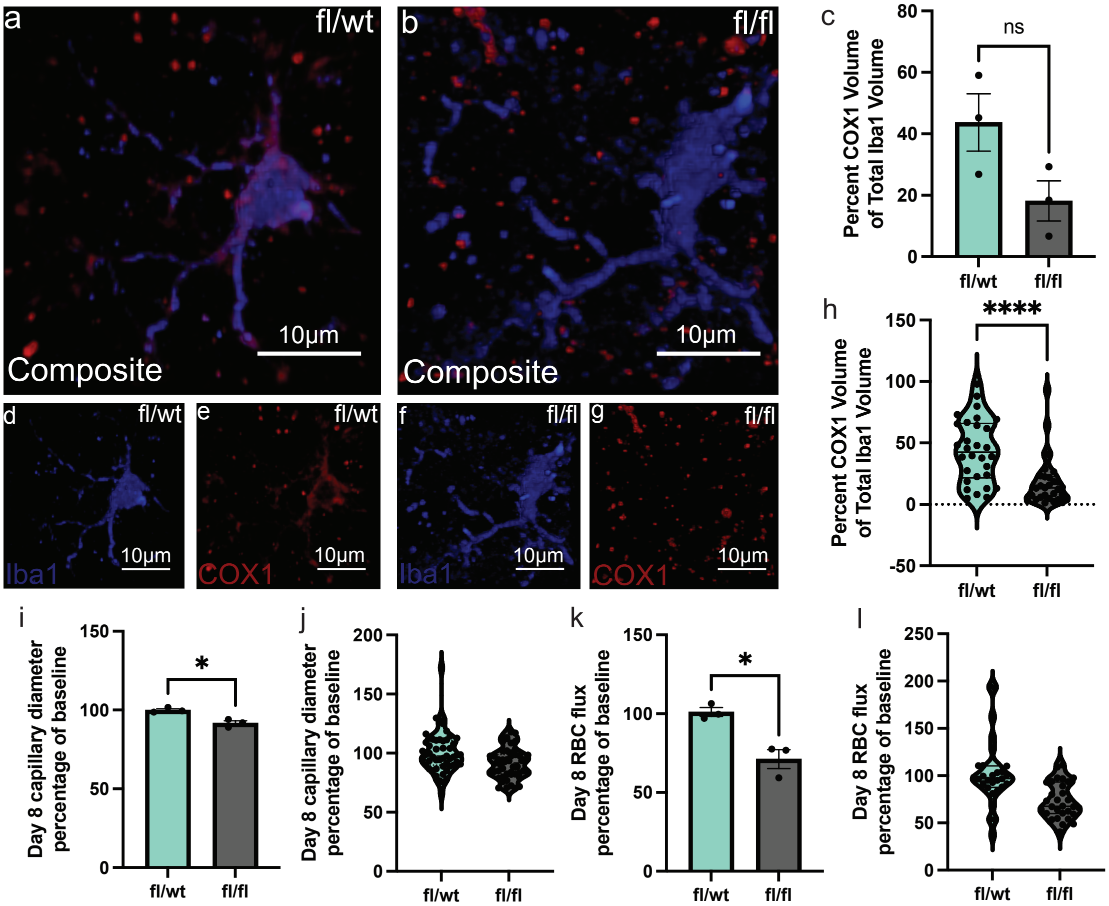

Supplementary Figure 8- COX1 protein expression is reduced by 50% following tamoxifen injection in TMEM119creERT2 x Ptgs1 fl/fl mice relative to TMEM119creERT2 x Ptgs1 fl/wt mice with subsequent reductions in capillary diameter and RBC flux as well. a-b) Volumetric 3D reconstruction showing COX1 expression (red) in an Iba1+ microglia (blue) in a Ptgs1 fl/wt mice, as in **a**, and a Ptgs1 fl/fl mice, as in **b**. **c)** bar graph comparing the percentage of COX1 volume occupying microglia Iba1 volume in Ptgs1 fl/wt and Ptgs1 fl/fl mice. n=30 microglia/3 mice. Two-tailed unpaired t-test, $p<0.0880$. **d-g)** Volumetric 3D reconstructions showing the Iba1 and COX1 channels in Ptgs1 fl/wt mice, as in **d** and **e** respectively, or the Iba1 and COX1 channels in Ptgs1 fl/fl mice, as in **f** and **g** respectively. **h)** Volcano plot showing the raw data used to generate the bar graph in **c**. n=30 microglia/3 mice. Mann-Whitney test, $p=0.0001$. **i**) bar graph quantifying the percentage of baseline values day 8 capillary diameter values are. n=51 capillaries/3 mice. Two-tailed unpaired t-test with Welch's correction, $p<0.0129$. **j)** volcano plot showing the raw data used to generate the bar graph in **i**. **k)** bar graph quantifying the percentage of baseline values day 8 RBC flux values are. n=29 capillaries/3 mice for Ptgs1 fl/wt mice and 30 capillaries/3 mice for Ptgs1 fl/fl

mice. Two-tailed unpaired t-test with Welch's correction, p<0.0225. **l)** volcano plot showing the raw data used to generate the bar graph in **k**. Source data are provided as a source data file. Data presented as mean ± s.e.m.

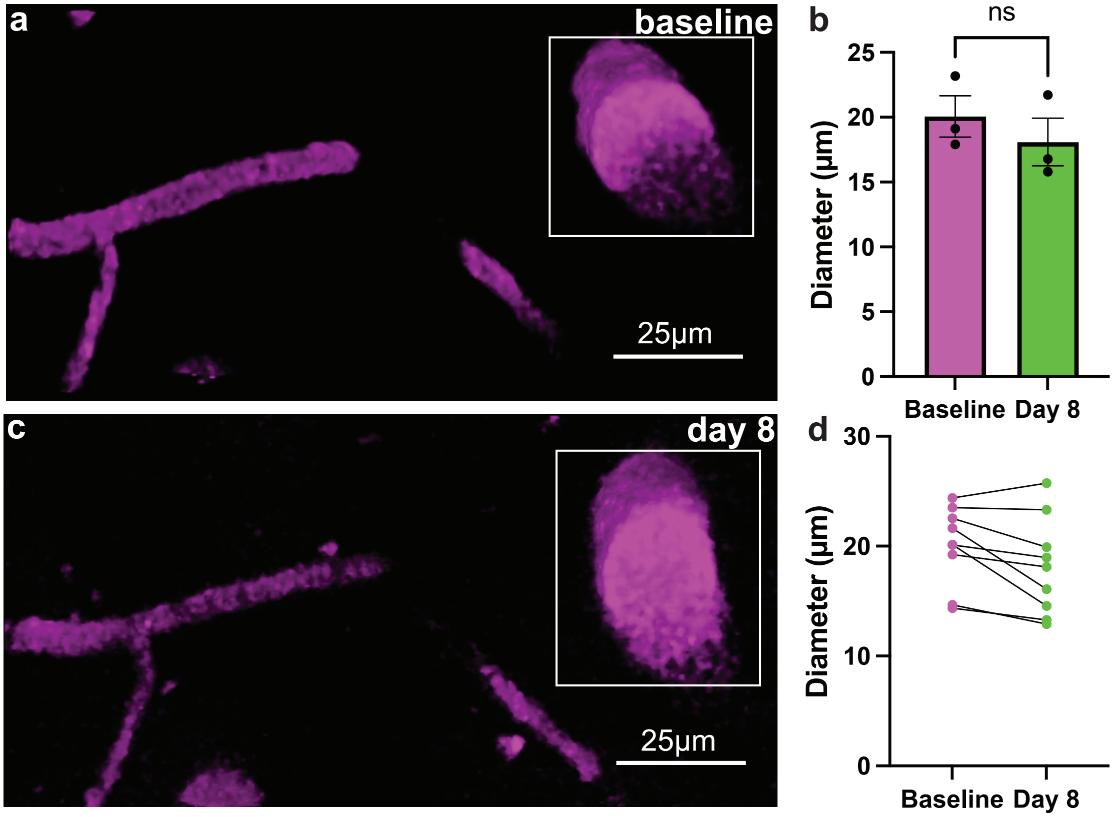

Supplementary Figure 9-No change in arteriole diameter following genetic ablation of microglial COX1. a,c) Volumetric 3D reconstruction showing an arteriole (magenta) at baseline (in white box) prior to tamoxifen administration, as in a, and 8 days after the last (fifth) tamoxifen administration, as in c. **b)** bar graph quantifying the arteriole diameter at baseline and at 8 days after the last tamoxifen administration. n=9 arterioles/3 mice. Two-tailed ratio paired t-test, p<0.1013. **d)** before-and-after plot showing the raw data used to generate the bar graph shown in b. LUTs have been adjusted to make arteriole diameter changes easier to visualize. Source data are provided as a source data file.
